# The Effect of Irrigation and Humic Acid on the Plant Yield and Quality of Sweet Basil (*Ocimum basilicum* L.) with Mulching Application under Semi-Arid Ecological Conditions

**DOI:** 10.3390/plants12071522

**Published:** 2023-03-31

**Authors:** Melike Sayarer, Zehra Aytaç, Mine Kürkçüoğlu

**Affiliations:** 1 Department of Field Crops, Faculty of Agriculture, Eskişehir Osmangazi University, Eskişehir 26160, Türkiye; 2 Department of Pharmacognosy, Faculty of Pharmacy, Anadolu University, Eskişehir 26470, Türkiye

**Keywords:** humic acid doses, irrigation water level, sweet basil, soil mulching, fresh yield, dry yield, essential oil, biostimulant

## Abstract

The adoption of suitable irrigation levels (IRL), humic acid doses (HAD) and soil mulching (SM) are important tools for improving the morpho-physiological and biochemical traits of medicinal and aromatic plants. *Ocimum basilicum* L. cultivated under four IRL: IRL 100 = 100% FC–IRL 75 = 75% FC–IRL 50 = 50% FC–IRL 25 = 25% FC and four HAD: HA 0 = 0.0 Lha^−1^–HA 10 = 10.0 Lha^−1^–HA 20 = 20.0 L ha^−1^–HA 40 = 40.0 L ha^−1^ were applied in order to evaluate morpho-physiological and biochemical traits under the ecological conditions of Eskişehir in 2016 and 2017. A second trial was conducted with black plastic soil mulch (SM) and compared with the control plots (CP) in 2016. The experiment was arranged in a randomized complete block design with split plots and three replications. The plant height (PH), fresh herb yield (FHY), dry herb yield (DHY), dry leaf yield (DLY), protein ratio (PR), and main essential oil compounds (MEOC) of *Ocimum basilicum* L. increased and the essential oil ratio (EOR) and essential oil yield (EOY) decreased with increasing IRL (IRL 100 and IRL 75). FHY (7268.3 and 7472.7 kg ha^−1^) and DLY (635.3 and 637.5 kg ha^−1^) increased with increasing HAD (HA 20 and HA 40) compared to the values of FHY and DLY at HA 0 (6852.6 and 587.0 respectively). The SM application at IRL 50 increased the PH between 8.8 and 13.5%, FHY 11.7 and 16.7%, DLY 22.5 and 29.2%, and at IRL 75 the EOY between 20.0 and 23.9% compared to CP. In addition, PH, FHY, DLY, and EOY were highest at HA 40 and HA 20. The MEOC (linalool, 1,8-cineole, and (E) – β-bergamotene) under SM were more pronounced at IRL 25 and IRL 50 compared to CP. HA particularly improved FHY, DLY, and the main essential oil compounds that can be considered plant biostimulants, which were defined by several studies and regulations.

## 1. Introduction

Irrigation significantly affects agricultural production in semi-arid ecological conditions, and an appropriate level of irrigation is required for the optimum yield and quality of plants. Due to the limited water resources, the use of drip irrigation systems, which provide the means to deliver water to the soil in small and frequent quantities at a relatively low cost, is a must in sustainable agricultural systems [1,2]. Drip irrigation is rather essential for the efficient use of the available freshwater resources which are crucial nowadays due to water constraints, especially in semi-arid ecological conditions. Therefore, determining different drip irrigation levels ensures appropriate water consumption [3]. To maintain a limited but appropriate level of drip irrigation, soil mulching is one of the sustainable agricultural methods to preserve the moisture of the soil and increase crop yield [4,5,6]. Soil mulching serves with efficient weed control, preserves water evaporation, produces cleaner crops, and protects them from diseases and pests [7,8,9,10,11]. In addition, it improves soil temperature, increases the soil’s organic carbon stock, and reduces greenhouse gas emissions [12]. These results are crucial in maintaining a sustainable agricultural system and are especially significant for annual plants such as basil (*Ocimum basilicum* L.), that are harvested 2–4 times in a single growing season.

Basil, belonging to the *Lamiaceae* family, is an annual spice plant that contains valuable essential oil and whose aromatic leaves are used fresh or dried as a flavoring agent for foods, beverages, and confectionery products. It holds economic significance and is cultivated throughout the world. Traditionally, in folk medicine, it has been used in the treatment of various ailments, such as headaches, coughing, diarrhea, constipation, warts, worms, and kidney malfunction. Externally, it can be used as an ointment for insect bites, and to treat acne [13,14,15,16,17]. The essential oil, mainly used in food industries and perfumery, also possesses antibacterial, antifungal, antiviral, and antiparasitic properties, and is linked to the presence of several bioactive compounds. Some of its compounds, such as 1,8-cineole, linalool, and camphor, are known to be biologically active [18,19,20,21,22]. 

Humic acid (HA) which is an essential component of healthy, productive soils, retains water, binds metal ions, sorbs organic solutes, photosensitizes soil reactions, stimulates plant growth, biotransforms toxic pollutants [23], and influences soil fertility through its effect on the water-holding capacity of soil [24]. Some studies have been conducted on the effects of HA and irrigation treatments on plant growth, and the essential oil quality of *Ocimum basilicum* L. [25,26] reported high values from high-level irrigation for the morpho-physiological traits conducted in field and glasshouse studies, and [27,28] reported positive relationships between the essential oil quality and water stress of basil plants. The increase in the essential oil ratio of sweet basil, with the decrease in irrigation levels, has been reported by many researchers [28,29]. HA application was effective on the growth and yield parameters of field-grown plants, and the highest values were obtained from the highest dose of humic acid [30]. 

Although humic acid is referred to as a plant biostimulant (PBS), which is becoming increasingly desirable for usage in sustainable agriculture production systems, the definition and categories of biostimulants in the world are yet to be clearly described. Efforts have been made to describe the principal categories of PBS in numerous studies. Reference [31] outlined two important categories for PBS such as humic acids and seaweed extracts, and their action on plants was proposed to be essentially hormonal. References [32,33,34] concluded that some major categories are widely recognized, covering both substances and microorganisms, and proposed several categories of substances that act as PBSs, such as humic and fulvic acids. The regulation of (EU) 2019/1009 on Fertilizing Products http://data.europa.eu/eli/reg/2019/1009/oj (accessed on 16 February 2023) defines PBS as a product that stimulates plant nutrition processes independently of the product’s nutrient content with the sole aim of improving one or more characteristics of the plant or the plant rhizosphere such as nutrient use efficiency; tolerance to abiotic stress; quality traits; and the availability of confined nutrients in soil or rhizosphere. PBSs are included in the product function categories of EU fertilizing products and are designed as microbial and non-microbial PBSs. In the United States, The Agriculture Improvement Act of 2018 (2018 Farm Bill) was passed into law and provides the first statutory language regarding PBSs. The bill describes a PB as a substance or micro-organism that, when applied to seeds, plants, or the rhizosphere, stimulates natural processes to enhance or benefit nutrient uptake, nutrient efficiency, tolerance to abiotic stress, or crop quality and yield https://bpia.org/solutions-provided-by-biological-products-biostimulants/ (accessed on 16 February 2023). The regulation of PBSs is dependent on the active components of the product and the claims made for it. PBSs could be considered a plant regulator under the Federal Insecticide, Fungicide, and Rodenticide Act, leading to its regulation by the Environmental Protection Agency. Alternatively, PBS can be regulated by agricultural state departments which regulate fertilizers, plant and soil amendments, and other products [35]. The EPA began a public comment period in November 2020 on the bill’s explanation of the differences between biostimulants and plant growth regulators, which is still under draft review to this day https://epa.gov/pesticides/draft-guidance-plant-regulators-and-claims-including-plant-biostimulants ( accessed on 16 February 2023). With the collaboration of the Biological Products Industry Alliance and The Fertilizer Institute, PBSs are placed into five compositional categories, one of these being “Complex Carbon-Based Biostimulants” which includes “mined natural deposits” such as humic acids, fulvic acids, and humin [36].

In Türkiye, the Regulation on the “Licensing of Pesticides and Similar Substances Used in Agricultural Protection” released the definition of “Pesticide-Like Substances” (Official newspaper, 17 February 1999, 23614). The term “plant activators” has been added under this article and the licensing of biostimulants takes place according to the aforementioned regulation (Official newspaper, 26 June 2002, 24797). Plant activators are defined as substances that have natural and/or chemical strengthening, resistance-enhancing, and soil structure-regulating properties and carry one or more of these properties which activate the natural defense system of plants, enabling them to make better use of nutrients, helping to protect them from stress conditions and similar external factors, positively affecting yield and product quality https://www.resmigazete.gov.tr/eskiler/2002/06/20020626.htm#12 (accessed on 17 February 2023). In addition, liquid humic acid and liquid fulvic acid are categorized as “products of other organic sources” under the Regulation of “Organic, organomineral fertilizers, soil regulators, products with microbial and enzyme constituents and other organic sources” (Official newspaper, 24 March 2014, 30341) https://www.resmigazete.gov.tr/eskiler/2014/03/20140329-5.htm (accessed on 17 February 2023).

The relationships between humic acid and irrigation levels with soil mulching on the yield and essential oil quality of basil plants under semi-arid ecological conditions have not been studied and discussed previously. It is vital for a sustainable agricultural system to preserve the water source, soil quality, and plant yield and quality simultaneously. In order to obtain these results, adopting suitable irrigation levels and humic acid doses through the use of soil mulching could provide significant information for growers and landholders. In addition, the results obtained from the use of humic acid in this study were compared and discussed with the definitions given by several regulations worldwide for the term “plant biostimulants”. All the aforementioned objectives demonstrate the absolute novelty of this study. The aim of this research was to study the effects of four irrigation levels and soil humic acid doses applied to soil (i) on the morpho-physiological and biochemical traits for two experimental years, (ii) with the application of plastic mulching on the morpho-physiological and biochemical traits of *Ocimum basilicum* L. under semi-arid ecological conditions, and (iii) to compare the results of the humic acid applications with the similarity of the previously determined definitions for “plant biostimulants”.

## 2. Results

### 2.1. The Influence of Irrigation and Humic Acid on the Morpho-Physiological and Biochemical Traits

#### 2.1.1. Morpho-Physiological Traits

Plant height (PH), fresh herb yield (FHY), dry herb yield (DHY), dry leaf yield (DLY), essential oil ratio (EOR), and protein ratio (PR) were significantly affected by different irrigation levels (IRL) in both years, whereas the number of branches per plant (NB) and chlorophyll value (CV) were significantly affected in 2017 only (Table 1 and Table 2). FHY and DLY were significantly affected by humic acid doses (HAD) in 2016 (Table 1) and essential oil yield (EOY) in 2017 (Table 2).

The mean values of PH, FHY, DHY, DLY, and PR in both years, as well as NB (9.39) and CV (46.2) in 2017, increased with the increasing level of IR. The highest mean values were obtained at IRL 100 (34.8–34.1 cm, 7625–7139 kg ha^−1^, 1124–979 kg ha^−1^, 652–566 kg ha^−1^ and 22.6–21.8%), and IRL 75 (35.5–34.9 cm, 7606–7065 kg ha^−1^, 1111–967 kg ha^−1^, 658–565 kg ha^−1^ and 23.2–21.0%) in both years. The mean values of EOR (0.48–0.60%) and EOY (3.1 L ha^−1^) decreased with the increasing level of IRL (at IRL 100).

The mean values of FHY and DLY increased with increasing HAD. The highest mean values were obtained at HA 20 (7268 kg ha^−1^ and 635 kg ha^−1^) and HA 40 (7473 kg ha^−1^ and 637 kg ha^−1^). Although NB was not significantly affected by the factors, it increased with increasing IRL (9.2 to 11.4) and HAD (9.8 to 11.0). Likewise, DHY (966 to 1043 kg ha^−1^) and PR (21.9 to 22.7%) also increased with increasing HAD.

The mean EOR of basil was affected by the irrigation levels at the *p* < 0.05 significance level. The values decreased with the increasing level of IRL in both years. The highest mean values were obtained at IRL 25 (0.66% and 0.75%) in both years and IRL 50 (0.70%) in 2017 (Table 1 and Table 2). 

The mean values of PR increased with the increasing level of IRL in 2017. The highest mean values were obtained at IRL 100 (21.8%) and IR 75 (21.0%) (Table 2). 

#### 2.1.2. Biochemical Traits

A total of 17 compounds were identified representing 90.2–97.4% of the EO (Table 3 and Table 4). In general, oxygenated monoterpene hydrocarbons (OMH), were present in the EO samples between 53.2 and 62.6%, whereas sesquiterpene hydrocarbons (SH) presented between 10.8 and 15.8% in both years, respectively (Table 3 and Table 4). Among the groups of chemical compounds, OMH mainly comprised linalool (49.6–59.5%), which was the main compound of the EO. SH was higher only in the second year at IRL 50 and IRL 25. The mean values of all EO groups except “others (O)” were generally higher in the second year compared to the first year at all IRLs. Some compounds belonging to “O” were higher in the first year because (Z)-methyl cinnamate and (E)-methyl cinnamate were not detected in the second year. The average values of the compounds of HAD were given under IRL groups. Particularly, the mean values of the compounds decreased with decreasing IRL. The mean values of IRL 100 and IRL 75 were higher than the values belonging to IRL 50 and IRL 25 (Table 3 and Table 4). 

**Figure 1:**
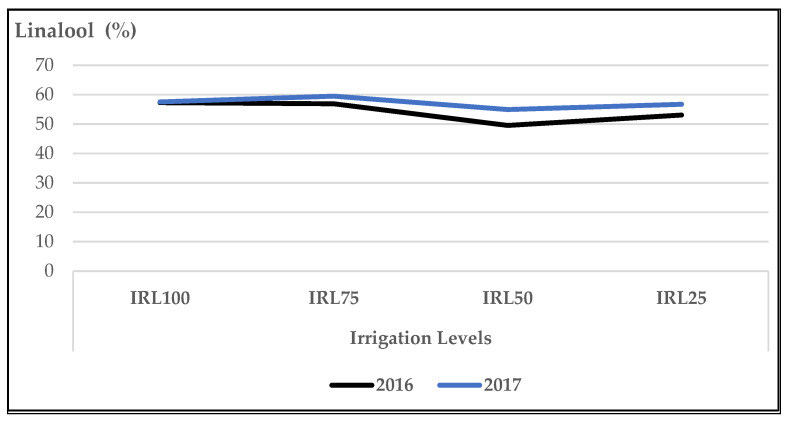
Mean values of linalool (%) at different IRLs of *O. basilicum* L. essential oil; IRL 100 = 100% FC; IRL 75 = 75% FC; IRL 50 = 50% FC; IRL 25 = 25% FC without SM.

**Figure 2:**
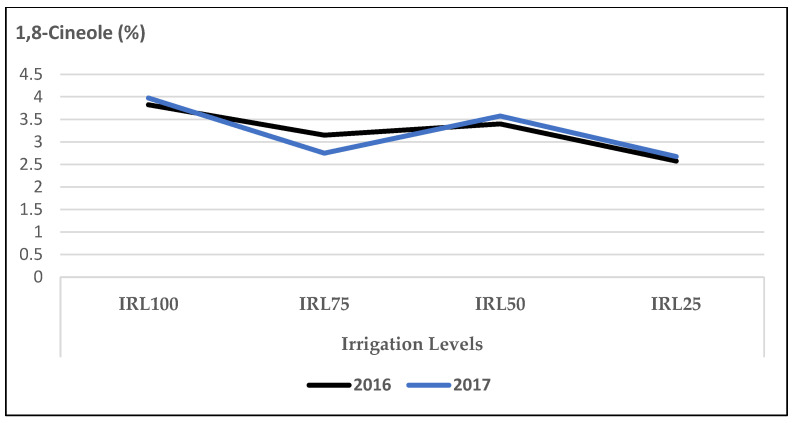
Mean values of 1,8-cineole (%) at different IRLs of *O. basilicum* L. essential oil; IRL 100 = 100% FC; IRL 75 = 75% FC; IRL 50 = 50% FC; IRL 25 = 25% FC without SM.

The mean IRL values of the main compounds “linalool and 1,8-cineole” were higher in the second year (Figure 1 and Figure 2). The mean contents of linalool at IRL 100 (57.3% and 57.5%) and IRL 75 (56.9% and 59.5%) were higher compared to IRL 50 (49.5% and 54.9%) and IRL 25 (53.1% and 56.7%) in both years, respectively (Figure 1). 

The mean values of 1,8-cineole at IRL 100 (3.8% and 3.9%) were higher compared to IRL 25 (2.6% and 2.7%) in 2016 and 2017, respectively (Figure 2). 

**Figure 3:**
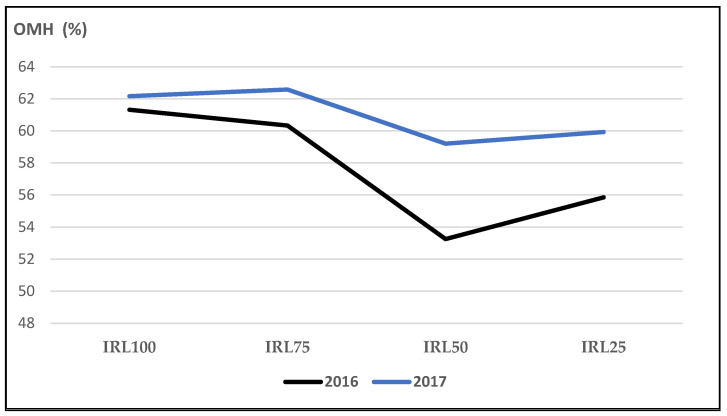
Mean values of OMH (%) at different IRLs of *O. basilicum* L. essential oil; IRL 100 = 100% FC; OMH: oxygenated monoterpene hydrocarbons, IRL 75 = 75% FC; IRL 50 = 50% FC; IRL 25 = 25% FC without SM.

OMH is included alongside linalool, camphor, and 1,8-cineole. The total amount of OMH was higher in the second year compared to the first year. The mean values at IRL 100 and IRL 75 were higher than IRL 50 and IRL 25 (Figure 3). 

**Figure 4:**
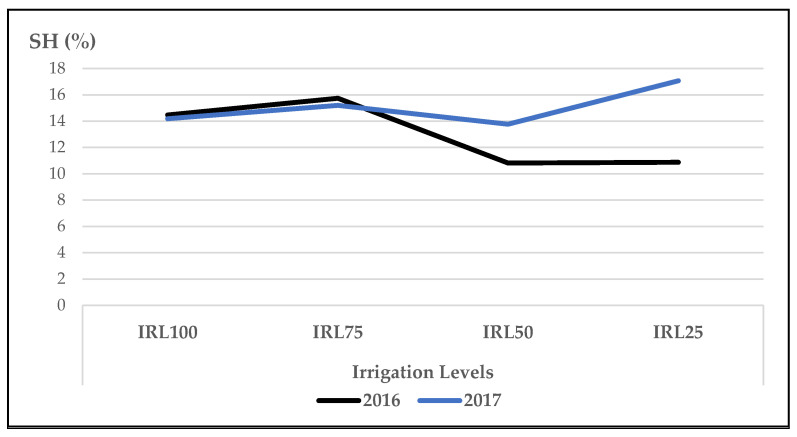
Mean values of SH (%) at different IRLs of *O. basilicum* L. essential oil; SH: sesquiterpene hydrocarbons; IRL 100 = 100% FC; IRL 75 = 75% FC; IRL 50 = 50% FC; IRL 25 = 25% FC without SM.

Sesquiterpene Hydrocarbons (SH) include (E)-β-bergamotene, α-guaiene/elemene, β-caryophyllene, germacrene D, γ-guaiene, bicyclogermacrene, and γ-cadinene. The total amount of SH was higher in the second year compared to the first year. The mean value at IRL 75 was higher than the previous IRLs in 2016, whereas it was highest at IRL 25 in 2017 (Figure 4). 

### 2.2. The Influence of Irrigation and Humic Acid on the Morpho-Physiological and Biochemical Traits with the Effect of Soil Mulching

#### 2.2.1. Morpho-Physiological Traits

PH, FHY, DLY, and EOY were significantly affected by different IRLs under soil mulching (SM) and control plots (CP) conditions. 

FHY was significantly affected by different HADs in both conditions, but DLY was affected only by control conditions. The interaction of IRL and HAD (IRL × HAD) was statistically significant for PH, FHY, and PR under SM conditions (Table 5). The mean values of the examined characteristics of *Ocimum basilicum* L. cultivated under different IRL and HAD conditions (2016) with SM conditions stated that the increasing doses of HA were in correlation with the increasing level of IR (Supplementary Table S1). Higher doses of HA worked efficiently with higher levels of IR for PH and FHY. The interaction effect was significantly important for PR but the IRL and HAD were not significant for this trait.

**Figure 5:**
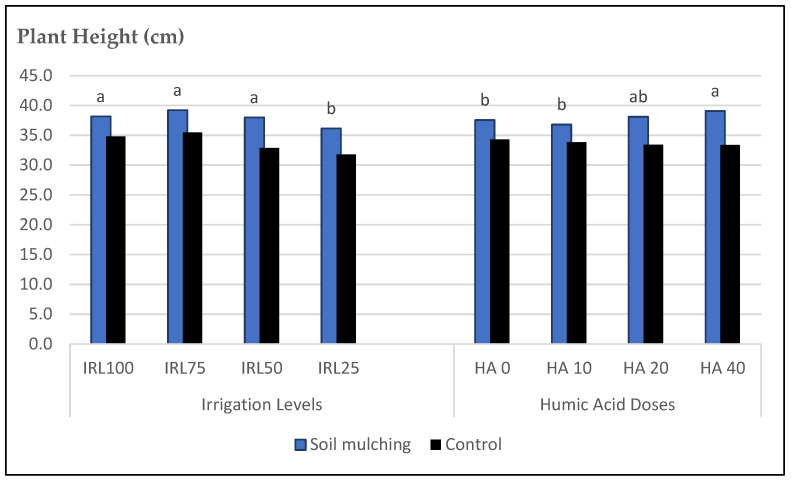
Mean values of PH of *O. basilicum* L. under soil mulching and control conditions. IRL 100 = 100% FC; IRL 75 = 75% FC; IRL 50 = 50% FC; IRL 25 = 25% FC; HA 0 = 0.0 Lha^−1^; HA 10 = 10.0 Lha^−1^; HA 20 = 20.0 Lha^−1^; HA 40 = 40.0 Lha^−1^.

The highest mean values for PH were obtained at IRL 75, IRL 100, and IRL 50 for soil mulching and CP. The mean values for SM plots were between 39.22 and 36.14 cm, but 35.46 and 31.78 cm for CP. The mulching application increased the PH between (+8.8 and 13.5%) compared to CP. The highest increase rate was obtained at IRL 50 (13.5%), whereas the lowest was obtained at IRL 100 (8.8%). For HAD, the mean values for soil mulching plots were between 39.07 and 36.81 cm. The highest mean values for PH were obtained at HA 40 (39.07 cm) and HA 20 (38.11 cm) from the SM plots. The mulching application increased the PH between (+8.1 and 14.6%) compared to CP. The highest increase rate was obtained from HA 40 (14.6%) and the lowest from HA 0 (8.6%) and HA 10 (8.1%) (Figure 5). 

**Figure 6:**
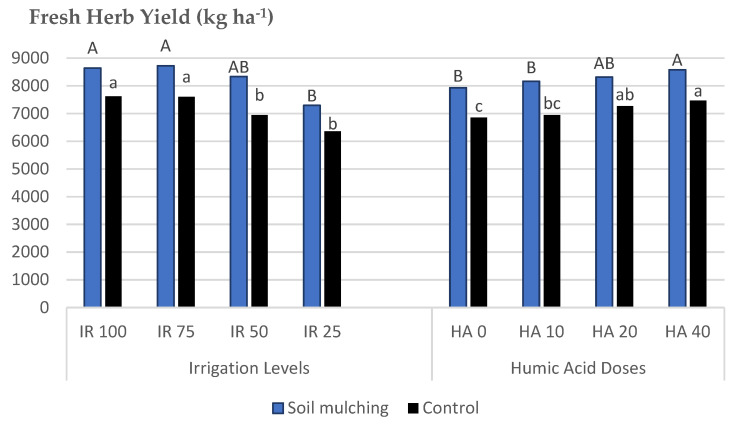
Mean values of FHY of *O. basilicum* L. under soil mulching and control conditions. IRL 100 = 100% FC; IRL 75 = 75% FC; IRL 50 = 50% FC; IRL 25 = 25% FC; HA 0 = 0.0 Lha^−1^; HA 10 = 10.0 Lha^−1^; HA 20 = 20.0 Lha^−1^; HA 40 = 40.0 Lha^−1^.

The highest mean values for FHY were obtained at IRL 75, IRL 100, and IRL 50 for SM and IRL 75 and IRL 100 for CP. The mean FHY values for SM plots were between 8727 and 7296 kg ha^−1^, but 7625 and 6364 kg ha^−1^ for CP. The mulching application increased the FHY between (+11.7 and 16.7%) compared to control plots. The highest increase rate was obtained at IRL 50 (16.7%), whereas the lowest was obtained from IR 100 (11.7%). Under soil mulching conditions, a higher FHY was obtained with only IRL 50, which has the highest FHY increase rate (16.7%), whereas without soil mulching, the highest FHY was obtained from IR 75 and IR 100. This spent the IRL up to 25–50%. For HAD, the mean values for SM plots were between 8580 and 7931 kg ha^−1^. The highest mean values for FHY were obtained at HA 40 (8581 kg ha^−1^) and HA 20 (8320 kg ha^−1^) from the SM plots. The SM application increased the FHY between (+12.6 and 14.9%) compared to CP. The highest increase rate was obtained at HA 10 (14.9%) and the lowest at HA 20 (12.6%). Under SM conditions, a higher FHY was obtained at HA 40 and HA 20, whereas without mulching, the HA applications were insignificant (Figure 6). 

**Figure 7:**
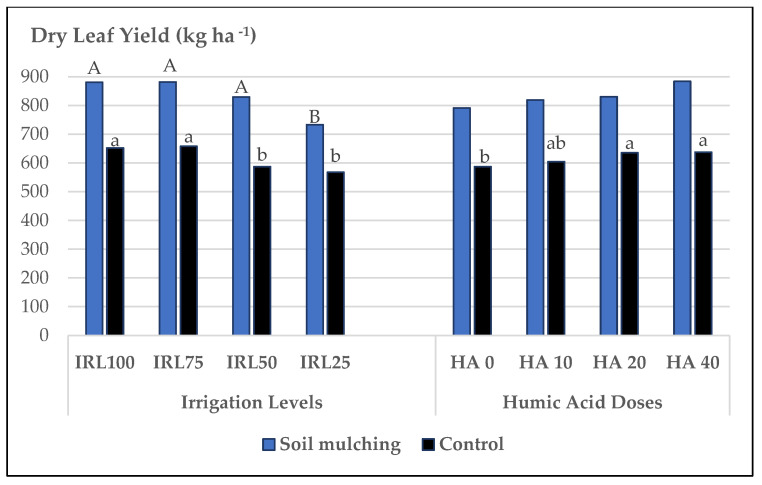
Mean values of DLY of *O. basilicum* L. under soil mulching and control conditions. IRL 100 = 100% FC; IRL 75 = 75% FC; IRL 50 = 50% FC; IRL 25 = 25% FC; HA 0 = 0.0 Lha^−1^; HA 10 = 10.0 Lha^−1^; HA 20 = 20.0 Lha^−1^; HA 40 = 40.0 Lha^−1^.

The highest mean values for DLY were obtained at IRL 75, IRL 100, and IRL 50 for SM and CP. The mean values for SM plots were between 882 and 733 kg ha^−1^, albeit 658 and 568 kg ha^−1^ for CP. The SM application increased the DLY between (+22.5 and 29.2%) compared to CP. The highest increase rate was obtained at IRL 50 (29.2%), whereas the lowest was obtained at IRL 25 (22.5%). For HAD, the mean values were not significantly different for SM plots, but the highest mean values for them were obtained at HA 40 (884 kg ha^−1^) and HA 20 (830 kg ha^−1^). The mean values for DLY for CP were significantly different, and the highest were obtained at HA 40 (638 kg ha^−1^) and HA 20 (635 kg ha^−1^). The SM application increased the DLY between (+23.5 and 27.8%) compared to CP. The highest increase rate was obtained at HA 40 (27.8%) (Figure 7).

**Figure 8:**
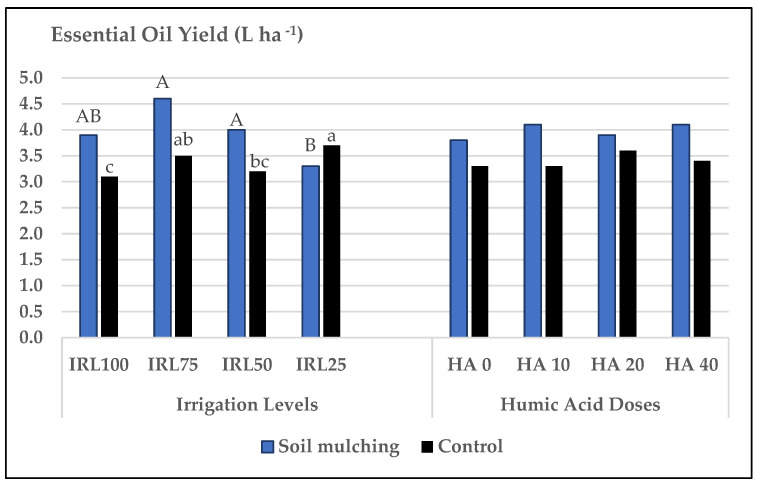
Mean values of EOY of *O. basilicum* L. under soil mulching and control conditions. IRL 100 = 100% FC; IRL 75 = 75% FC; IRL 50 = 50% FC; IRL 25 = 25% FC; HA 0 = 0.0 Lha^−1^; HA 10 = 10.0 Lha^−1^; HA 20 = 20.0 Lha^−1^; HA 40 = 40.0 Lha^−1^.

The highest mean values for EOY were obtained at IRL 75 and IRL 50 for SM and from IRL 25 and IRL 75 for CP. The mean values for SM plots were between 4.6 and 3.3 L ha^−1^, but 3.7 and 3.1 L ha^−1^ for CP. The highest increase rate of the SM application was obtained at IRL 75 (23.9.2%), whereas the lowest was obtained at IRL 50 (20.0%) compared to CP. IRL 25 decreased the EOY of SM plots (−12.1%). For HAD, the mean values were not significantly different between SM and CP, but the highest mean values were obtained at HA 40 and HA 20 for both applications (Figure 8). 

The summary of the first and second trials is displayed in Table 6. As is seen in the table, the mean values of the first trial belonging to the first year were higher than those of the second year. The results of the soil mulching application were higher for PH, FHY, DHY, DLY, and EOY, as mentioned below. 

#### 2.2.2. Biochemical Traits

Analysis of the essential oil components demonstrated that the ratio of “linalool, I-β-bergamotene, β-caryophyllene, germacrene D and T-cadinol” generally increased at HA 40 (61.0%, 5.0%, 2.4%, 3.2%, and 5.1%), and this increase was more pronounced compared to HA 0 (50.6%, 3.1%, 0.9%, 2.4%, and 5.1%) obtained at IRL 100 under control conditions, respectively (Table 7). In SM conditions, the ratio of linalool and (E) β-bergamotene also generally increased with increasing doses of HA at all IRLs. This increase was more pronounced at the dose of HA 40 (62.1% and 5.4%) obtained at IRL 50, respectively, compared to HA 0 (60.7% and 5.2%). In addition, these main compounds increased at the dose of HA 40 (61.6% and 5.1%) compared to HA 0 (59.3% and 3.6%) obtained at IRL 25, respectively (Table 8). 

The ratios of the main compounds, linalool, 1,8-cineole, and (E)-β-bergamotene, increased with the decrease in the IRL in SM conditions, and this increase was more pronounced at IRL 25 compared to CP. 

**Figure 9:**
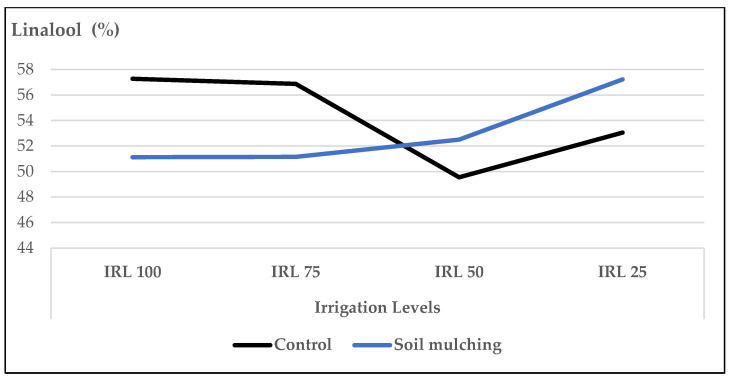
Mean values of linalool (%) at different IRLs of *O. basilicum* L. essential oil under soil mulching and control conditions; IRL 100 = 100% FC; IRL 75 = 75% FC; IRL 50 = 50% FC; IRL 25 = 25 °C.

The mean contents of linalool at IRL 50 (52.5%) and IRL 25 (57.2%) were higher compared to the CP at the same IRLs (49.5% and 53.05%), respectively. On the other hand, the mean contents of linalool at IRL 100 (57.3%) and IRL 75 (56.8%) in CP conditions were higher compared to the SM conditions at the same IRLs (51.1% and 51.15%), respectively (Figure 9).

The mean contents of (E)-β-bergamotene at IRL 100 (4.4%), IRL 75 (3.8%), IRL 50 (3.8%), and IRL 25 (4.2%) were higher compared to the CP at the same IRLs (4.1%, 3.2%, 2.9% and 2.5 %), respectively.

**Figure 10:**
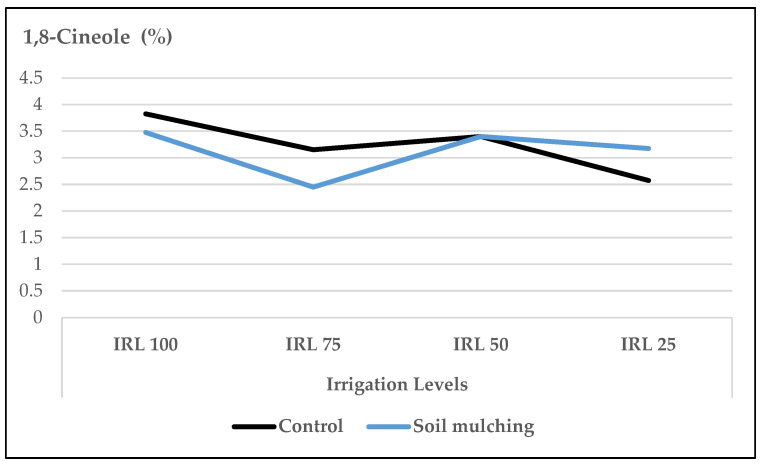
Mean values of 1,8-cineole at different IRLs of *O. basilicum* L. essential oil under soil mulching and control conditions; IRL 100 = 100% FC; IRL 75 = 75% FC; IRL 50 = 50% FC; IRL 25 = 25%.

The mean contents of 1,8-cineole at IRL 50 (3.5%) and IRL 25 (3.2%) were higher compared to the CP at the same IRLs (3.4% and 2.6%), respectively. On the other hand, the mean content of 1,8-cineole at IRL 100 (3.8%) and IRL 75 (3.1%) in CP conditions were higher compared to the SM conditions at the same IRLs (3.5% and 2.5%), respectively (Figure 10).

**Figure 11:**
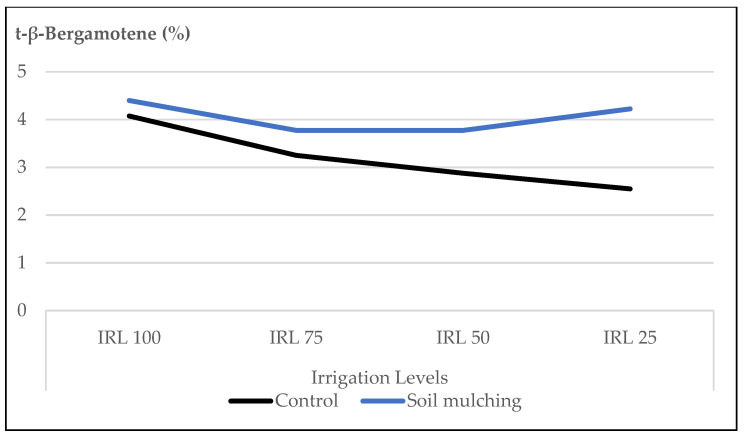
Mean values of (E)-β-bergamotene (%) at different IRLs of *O. basilicum* L. essential oil under soil mulching and control conditions; IRL 100 = 100% FC; IRL 75 = 75% FC; IRL 50 = 50% FC; IRL 25 = 25% FC.

The mean contents of (E)-β-bergamotene at IRL 100 (4.4%), IRL 75 (3.8%), IRL 50 (3.8%), and IRL 25 (4.2%) were higher compared to the CP at the same IRLs (4.1%, 3.2%, 2.9% and 2.5%), respectively (Figure 11). 

## 3. Discussion

### 3.1. The Influence of Irrigation and Humic Acid on Morpho-Physiological and Biochemical Traits

#### 3.1.1. Morpho-Physiological Traits

The effect of irrigation on PH, FHY, DLY, EOR, and PR was found to be statistically significant for both years (Table 1 and Table 2). IRL 100 and IRL 75 showed the highest increase in morpho-physiological characters. This could be due to the increased vegetative growth. Similarly, a field study on purple basil with different IRLs reported the highest PH, FHY, DHY, and DLY from the highest IRL (125) in both years. The mean values of yield increased with the increasing level of IR [25]. Likewise, ref. [28] found higher total fresh and dry yields from IRL 75. Fresh and dry weights decreased at IRL 50 and IRL 125. The second was due to excessive water amount which was also injurious. In our study, IRL 100 caused no injury and was in the same statistical group as IRL 75 (Table 1 and Table 2). Ref. [37] stated that the total herbage and oil yields increased significantly with the increase in the IRL from 0.25 to 0.75 IW/CPE ratio. In fact, it was demonstrated that the water amount has a crucial role in plant growth. Plants grow primarily by increasing cell water content. The enlargement occurs because solute concentrations are high enough inside the cells to extract water osmotically from the surroundings. As a result, the pressure in the cells (turgor) rises and extends the walls irreversibly, enlarging the cell compartment. The dependency of these processes on water causes growth to respond to the supply of water in the soil [38]. Water stress causes a significant decrease in plant height, the number of inflorescence branches, the number of leaves, leaf area, the fresh and dry weight of leaves and stems, relative water content, and chlorophyll concentration in sweet basil plants. Some studies showed higher values for most of the traits that were observed at IRL 80 [26,39,40]. In addition, the increase in the yield and yield characters of the highest IRL could be due to the increase in the chlorophyll value (CV) and, consequently, photosynthesis [28]. This may be explained by the higher CVs which were obtained at IRL 100 and IRL 75. Ref. [24] reported the generally insignificant effect of irrigation on the CVs with the exception of one cultivar in which the water supply increased the CV significantly. They concluded that a higher CV does not necessarily induce an increase in chlorophyll content. Likely, they also reported higher PH and fresh and dry mass with irrigation compared to the control plants. According to the CV, the biostimulant applications of humic and fulvic acids caused higher values of chlorophyll in the case of the rain-fed basil plants compared to the plants which were fully irrigated [41]. 

The mean values of EOR in this study increased with decreasing levels of IRL in both years. The highest mean values were obtained at IRL 25 and the lowest from IRL 100 in 2016 and 2017, respectively (Table 1 and Table 2). Ref. [25] found the highest EOR at the lowest IRL (50). The increase in the EOR due to the decrease in the IRL has also been reported by references [23,24,26,28,29]. In fact, drought stress might induce the closure of stomata. This may result in a reduction in CO_2_ uptake and biomass production. The biosynthesis may result in the accumulation of secondary metabolites [42,43]. 

Higher ratios of PR were obtained at IRL 100 and IRL 75 in the first year. Similarly, [28] reported a high ratio of PR obtained at IRL 100 and IRL 75 for *Ocimum basilicum* L. and *Ocimum americanum* L. in two seasons. On the other hand, ref. [44] reported an increase in the nitrogen content of the leaves of *Ocimum gratissimum* as a result of water stress. This may cause the mobilization of nitrogen to the leaves for the synthesis of proteins to maintain the plants to resist the effects of drought. This result was different from our findings and could be due to the differences in the basil species or the alleviation of the negative conditions of the low IRLs with the help of HAs. It is known that HAs increase the permeability of plant membranes and intensify the enzyme systems of plants. They accelerate cell division, show greater root development, and decrease stress deterioration [45]. Ref. [46] found higher leaf nitrogen concentrations from the plants treated with HA compared to the control plants.

The mean values of FHY and DLY increased with increasing HAD. The highest mean values were obtained at HA 20 and HA 40. FHY and DLY were significantly affected by HAD, particularly in the first year, being in accordance with [28,41]. PH and dry weight were increased with increasing doses of HA [47]. Ref. [30] stated that the application of HA on the growth and yield parameters of field-grown basil plants was effective, and they observed the best growth and yield with the highest HAD. A field study conducted with coriander reported increased plant growth, plant height, biological yield, and grain yield with the application of HA [48]. Ref. [9] reported an increased cotton stem diameter, boll number per plant, plant height, and leaf area index compared to control plants. The researcher recommended higher doses of potassium humate which serves water conservation in saline soils under SM. HA probably affects the activity of the Rubisco enzyme, which has a photosynthetic potential, absorbs macro- and micronutrients, increases cell membrane permeability, and eventually improves yield [49]. As stated above, the HA used in this study improved FHY and DLY at HAD 20 and HAD 40, and yield values were significantly affected by HADs. Based on these findings, humic acid can be defined as a plant biostimulant, as defined by the 2018 Farm Bill https://bpia.org/solutions-provided-by-biological-products-biostimulants/ (accessed on 16 February 2023).

#### 3.1.2. Biochemical Traits

In our study, the mean values of the main compound, linalool, at IRL 100 and IRL 75 were higher compared to those at IRL 50 and IRL 25 in both years (Figure 1). Similarly, 1,8-cineole was highest at IRL 100 (Figure 2). Ref. [24] stated that regular water supply increased the ratio of linalool and 1,8-cineole for some basil cultivars, but most of the changes were not significant. On the other hand, ref. [25] reported positive effects on the essential oil composition under water stress. Similarly, ref. [27] stated that linalool and methyl chavicol increased as water stress increased, and [28] stated that higher linalool and 1,8-cineole contents were obtained at IRL 50. The total amount of IMH was higher in the second year compared to the first year. This was because of the high amounts of components in the second year. The mean value of OMH at IRL 100 was higher than the previous IR levels (Figure 3). However, ref. [24] reported a decrease of 22% from the monoterpenes of the cultivar ‘Kasia’. The total amount of SH was higher in the second year compared to the first year. This was because of the high amounts of the components in the second year, particularly at IRL 50 and IRL 25. The mean value at IRL 75 was higher than the previous IRLs in 2016, whereas it was highest at IRL 25 in 2017 (Figure 4). Ref. [24] reported a general increase in SH with the increase in water supply compared to the control plants, but it was insignificant. In addition, ref. [27] found a lower relative proportion of sesquiterpenes with water stress. Essential oil production has been reported to be associated with plant ontogeny, the site of oil production, photosynthesis, photoperiodic modulation, the effect of light quality, seasonal and climatic variations, nutritional relationships, plant growth regulators, and abiotic stresses (moisture, salinity, and temperature) [50].

### 3.2. The Influence of Irrigation and Humic Acid on Morpho-Physiological and Biochemical Traits with the Effect of Soil Mulching

#### 3.2.1. Morpho-Physiological Traits

This study was conducted in semi-arid ecological conditions where water conservation is a crucial task for sustainable crop production. The annual rainfall averages are limited between 360 and 396 mm and evaporation rates are high [51]. Some tools, i.e., mulch, utilized irrigation water consumption and evaporation rates. The growth and habits of the basil plants which grew on the mulching material were stronger, and the plant height was higher. The mulching material may cause a special microclimate which decreases the differences in the soil temperature between night and day. At night, the temperature of the soil did not decrease because of the black-colored mulching material which has the potential to preserve heat and not reflect it. Similar results were obtained from the early growth stages of maize plants cultivated under a mulched drip irrigation system in a low-temperature environment [4]. In addition, the evaporation was decreased, and the plants were less affected by sun exposure during the hottest hours of the day. Ref. [10] conducted a field experiment with sweet basil under dry and hot tropical climate conditions to determine the effects of organic and synthetic mulches on the yield of sweet basil under a drip irrigation system. The research resulted in lower irrigation water usage, weed biomass, and higher soil temperature compared with bare soil. As a result, for favoring initial plant growth, they recommended black mulching materials in temperate climates where soil temperatures are low. This would be more effective in elevating the soil temperature rather than using organic mulches which decreased the soil temperature, particularly in the early spring.

The mulching application in this study led to an increase in the PH compared to CP. The highest increase rate was obtained at IRL 50. This result was important to obtain higher PH under SM conditions with only IRL 50, whereas, at CP, the highest PH was obtained at IRL 100 and IRL 75. This is an important level of water conservation which means obtaining a high PH with only irrigation at 50% field capacity could spend 25–50% of water consumption compared to the CP (Figure 5). Likewise, ref. [40] stated that mulching was helpful for growing plants efficiently under water-deficient conditions. For HAD, the mulching application increased the PH compared to the CP. The highest increase rate was obtained at HA 40, whereas the lowest values were obtained at HA 0 and HA 10. Without soil mulching, it was observed that the HA applications were not significantly effective (Table 5). The HA worked well and was efficient under mulching material because the moisture of the soil was held by means of mulching and in addition, by means of the application of HA, which worked efficiently due to its water holding capacity. Ref. [45] stated that HA significantly reduced water evaporation and increased water usage and water holding capacity by plants, especially in arid and sandy soils. Ref. [40] reported that drought stress had a significant decreasing effect on all experimented traits; however, the use of rhizobacteria with HA yielded a positive effect on evaluated characteristics in both stress and non-stress conditions. Likewise, ref. [11] reported that soil under black fabric mulch obtained higher soil moisture compared to the previous mulching materials.

The application of SM increased the FHY compared to CP. The highest increase rate was obtained at IRL 50. This result was important to produce higher FHY under SM conditions with only IRL 50, whereas at CP, the highest FHY was obtained at IRL 75 and IRL 100. This was an extremely significant difference that conserved up to 25–50% of water compared to the CP (Figure 6). On the contrary, ref. [11] stated that under SM, the herbal yield of rainfed mint plants was significantly lower than that of the irrigated plants. This difference could be due to the high evaporation rates of the soils, which varied under different ecological conditions. Likewise, ref. [10] reported that plots under black plastic mulch produced higher fresh plant and fresh leaf weight compared to the plots with black fabric mulch and control plots. Under SM conditions, higher FHY was obtained at HA 40 and HA 20, whereas without mulching, it was seen that the HA applications were not significant (Table 5). Potassium humate significantly increased cotton stem diameter, boll number per plant, plant height, and leaf area index compared to the control under the film-mulched drip irrigation system [8].

DLY is a significant trait because dry spice material and mainly the essential oil are produced from the leaves of the plant. Generally, the higher the DLY, the higher the EOY. Moreover, higher DLY contributes to the economic advantages of the crop and is mainly dependent on FHY. The SM application increased the DLY compared to CP. Similar to the FHY findings, the highest increase rate was obtained at IRL 50 and the lowest at IRL 25, whereas at CP, the highest DLY was obtained at IRL 100 and IRL 75 (Figure 7). When it comes to HAD, the SM application was not significant, but it increased the DLY compared to CP. The highest increase rate was obtained at HA 40. The mean values for DLY for CP were significantly different, and the highest were obtained at HA 40 (Figure 7). There are not any references about the effects of soil mulching, irrigation, and humic acid on the morpho-physiological traits of *Ocimum basilicum* but a study stated that organic mulching enhanced the growth and quality of *Cucurbita pepo* L. grown on silty loam soil [52].

EOY is a trait related to EOR and DLY. The highest increase rate of the SM application was obtained at IRL 75, whereas the lowest was obtained at IRL 50 compared to CP. This is associated with the increase in EOR at low IRLs, and with the increase in DLY, at higher IRLs (Figure 8). Ref. [11] reported that the straw mulching treatments increased the EO content (+16%) of the mint plants without irrigation compared to the plants which were irrigated under mulching conditions.

#### 3.2.2. Biochemical Traits

Under SM conditions, the ratio of linalool and (E)-β-bergamotene increased with increasing doses of HA, particularly at IRL 50 and IRL 25. The increase in the aforementioned essential oil compounds at IRL 50 was more pronounced at the dose of HA 40 compared to HA 0, respectively. In addition, these main compounds at IRL 25 increased at the dose of HA 40 compared to HA 0, respectively (Table 8). Under control conditions, the ratios of linalool, (E)-β-bergamotene, β -caryophyllene, germacrene D, and T-cadinol generally increased at HA 40 compared to HA 0, and this increase was more pronounced at IRL 100 (Table 8). As stated above, the HA used in this study increased the main essential oil compounds, particularly at HAD 40 at IRL 50 under SM conditions. Because of these results, humic acid can be considered a plant biostimulant, as defined by the regulation of (EU) 2019/1009, in which the definition stated the improvement of quality traits http://data.europa.eu/eli/reg/2019/1009/oj (accessed on 16 February 2023). Research results that compare the effect of SM with IRL and HA applications do not exist; however, in a study, HA applications increased the essential oil compounds of *Ocimum basilicum* [53]. Most of the essential oil compounds belong to terpenoids. The accumulation of terpenoids is directly affected by the potential of photosynthesis. In addition, CO_2_ and glucose are the initial precursors in forming essential oils [54]. 

When the effect of IRL is highlighted solely, the main compounds linalool, 1,8-cineole, and(E)-β-bergamotene were more pronounced at IRL 25 compared to CP under SM conditions. As is seen in Figure 9, Figure 10 and Figure 11, the use of SM at IRL 25 produced higher linalool, 1,8-cineole, and (E)-β-bergamotene percentages compared to CP (bare plots). The CP was highest at IRL 100 and IRL 75. There are no data to compare the effect of SM with the IRL belonging to the basil plant, but ref. [11] reported significantly higher percentages of limonene and 1,8-cineole from rainfed spearmint plants without a mulching application. However, the highest percentage of carvone was obtained from the irrigated and mulched plots. These findings were also stated above in Figure 1, Figure 2 and Figure 3. This is an important level of water preservation, which means a high-quality essential oil can be obtained (higher linalool, 1,8-cineole, and (E)-β-bergamotene) with irrigation at only 25% field capacity, resulting in spending 50–75% of water consumption under soil mulching in semi-arid ecological conditions. 

## 4. Materials and Methods

### 4.1. Site Conditions

Field experiments were carried out during two growing seasons (2016 and 2017) at the Faculty of Agriculture (Eskisehir Osmangazi University, Türkiye. N 39°48′, E 30°31′, altitude 789 m). 

The research area is characterized by a continental semi-arid climate with a long-term average annual rainfall of about 353.5 mm and an average annual temperature of 12.87 °C. Some meteorological data of the location during the vegetation were obtained from the weather station at Eskisehir (Table 9). The average air temperature (16.9–16.4 °C) was similar during the growing seasons in both years. The total rainfall amount was slightly higher (221.6 mm), especially in April, May, June, and August 2017 compared to the same months in 2016 and the long term. The humidity rates in both years were similar (Table 9). 

Soil samples (0–30 cm) were taken at the beginning of the vegetation period in both years. They were dried, sieved through a 2 mm stainless sieve, and analyzed for pH (1:2.5 soil/water), electrical conductivity (EC, 1:2.5 soil: water) [55], lime (Scheibler calcimeter), organic matter [56], available K (1N ammonium acetate, pH 7), available P (sodium bicarbonate method) [57], and texture (hydrometer method). The Fe, Cu, Mn, and Zn concentrations were analyzed [58] with an atomic absorption spectrometer (Analytik Jena novAA 350, Jena, Germany). The main chemical properties and heavy metal contents of the soil are included in Table 10. The soil of the experimental area in both years was alkaline and moderately calcareous with low contents of organic matter and salt concentration. A loamed textured soil with insufficient contents of P_2_O_5_ and Zn, moderately sufficient contents of K_2_O and Fe, and sufficient contents of Mn and Cu were obtained. The organic matter and available P_2_O_5_, Fe, Zn, Mn, and Cu contents of the research area in the second year were lower than those in the first year (Table 10).

The water quality is primarily dependent on the water salinity hazard as measured by electrical conductivity (EC dS/m at 25 °C). The irrigation water used in the research was between 0.76 and 1.5 dS/m, which was between “none and moderate limitations for use as irrigation water” based upon conductivity, stated as “some limitations for use” [59]. The quality of the irrigation water is given in Table 11. The analyses were conducted at the Transitional Zone Agricultural Research Institute in Eskişehir.

### 4.2. Field Experiments

Seeds of *Ocimum basilicum* L., belonging to a population and originating from Malatya, Türkiye, obtained from Süleyman Demirel University, the Department of Field Crops, were used as material. The seeds were sown into multi-pots with a mixture of sand, mulch, and manure (1:1:1) in April 2016 and 2017. The seedlings were transplanted to the research field in June at an inter-row spacing of 40 cm and an intra-row spacing of 20 cm. The experiment was arranged in randomized complete blocks as a split-plot design with three replications. The main plots of the experiment consisted of four IRLs. The subplots consisted of four HADs. The second trial was established under SM application with the same main (IRL) and sub-plot (HAD) design that was conducted in the first year. The plants were fertilized with 100 kg N ha^−1^ and 80 kg P ha^−1^ using triple super phosphate and ammonium nitrate applied following planting [60]. After the seedlings were established, the plants were cut 15 cm from the soil surface to maintain uniformity. This first cut was important to continue the experiments with homogenous plant growth. The weeds were controlled via hand weeding. No pesticides were utilized. 

A drip irrigation system was used in the field experiments. The lateral lines were 16 mm in diameter and were arranged along each row of the plot. The emitter space was 20 cm, the distance between the laterals was 40 cm, and in line pressure-controlled emitters with (the flow rate) 4 L h^−1^ discharge at 1 atm operating pressure. Irrigation (IR) was provided by taking the effective root depth of 30 cm of the basil plant because of its shallow root structure which does not exceed 45 cm in depth [61,62]. The IR amounts were calculated by measuring the amount of water that was evaporated in the Class A evaporation vessel. Irrigation, based on full IR, was started when 50% of the water was consumed at 30 cm soil depth held as the field capacity (FC). Since a total of 39 plants were planted in each plot, 3 rows and 13 plants in each row, 3 lateral lines were placed into each plot. In total, 156 L of water h^−1^ was provided with 39 drippers under full irrigation conditions, which was 100% FC at 4 L h^−1^. 

The IRLs were calculated depending on the amount of water evaporated daily. Irrigation was carried out every 4 days in June and September and every 3.5 days in July and August in both years.

Four irrigation levels (IRL): IRL 100 = 100 % FC

IRL 75 = 75% FC

IRL 50 = 50% FC 

IRL 25 = 25% FC 

Four humic acid doses (HAD): HA 0 = 0.0 Lha^−1^

HA 10 = 10.0 Lha^−1^

HA 20 = 20.0 Lha^−1^

HA 40 = 40.0 Lha^−1^

HA was applied from a liquid humic acid source (TKI-Humas) produced from leonardite [63]. This product (Table 12) was obtained by dissolving treated natural leonardite in solid form with potassium hydroxide. Detailed information about HA analyses is written in a previous study [64]. The HA was applied once after the first cut in both years.

Harvesting was performed manually at the beginning of September 2016 and 2017. The harvest cut height was 7–10 cm from the soil surface [65]. The harvest of the plants started at the beginning of the blooming stage [66]. 

Field trials were conducted in 2016 and 2017 using four different HADs and IRLs. Plant height, the number of branches per plant, fresh herb yield, dry herb yield, dry leaf yield, chlorophyll value, essential oil rate, essential oil yield, and protein content were evaluated. An experiment using plots with black plastic soil mulch (SM) was conducted in 2016 and compared with the plots without mulch; control plots (CP). The black plastic mulch was a 0.025 mm-thick polyethylene plastic. For both trials, 40-day-old seedlings were transplanted into three rows for each plot using a plant spacing of 20 cm in rows that were 40 cm apart.

### 4.3. Experimental Procedure

#### 4.3.1. Morpho-Physiological Traits

Plant height (cm), the number of branches per plant, fresh herb yield (kg ha^−1^), dry herb yield (kg ha^−1^), dry leaf yield (kg ha^−1^), dry herb rate (%), dry leaf rate (%), and chlorophyll value (SPAD-502 Konica, Minolta, Tokyo) were investigated for two experimental years in 2016 and 2017. The measurements were taken prior to each year during the beginning of flowering. Fresh herb yield was weighed after the harvest. In total, 500 g of fresh herbs per plot was wilted at room temperature for a week and then dried at 3 °C for 24 h. The dry herb yield was weighed after the drying process. In order to find the dry leaf yield, the leaves and flowers were separated from the stems and then weighed. 

#### 4.3.2. Essential Oil Content

The harvested plants were dried, separated into individual leaves, and stored in paper sacks until distillation. In total, 15 gr of dried plant parts were distilled by the Clevenger apparatus for two hours. The essential oil rate (%) of the plants was found by a volumetric method (mL/100 g) [67]. The average content of essential oil for each application was obtained from three repetitions. The essential oil yield was calculated using the dry leaf yield (kg ha^−1^) and the essential oil rate (%).

#### 4.3.3. Composition of Essential Oil

The composition of essential oils was analyzed by using an Agilent GC-MSD system (capillary GC/FID and GC/MS).

GC/MS conditions: HP-Innowax FSC column (Hewlett-Packard-HP, U.S.A.) (60 m × 0.25 mm i.d., with 0.25 μm film thickness), and helium was used as a carrier gas (0.8 mL/min). The split ratio was 40:1 flow, and the split was adjusted at 40 mL min^−1^. The injector temperature was 250 °C. Mass spectra were taken at 70 eV with a mass range of *m/z* 35–450. The GC oven temperature was kept at 60 °C for 10 min and programmed to be 220 °C at a rate of 4 °C/min and kept constant at 220 °C for 10 min and then programmed to be 240 °C at a rate of 1 °C/min [68].

GC-FID conditions: An Agilent 6890 N GC system fitted with an FID detector was used and set at a temperature of 300 °C. The relative percentage of the compounds was calculated from FID chromatograms by using Agilent Chem Station LC B.04.03 software with a peak integration process.

The identification of essential oil components was performed through a comparison of their mass spectra with those in the Baser Library of Essential Oil Constituents, Wiley GC/MS Library, Adams Library, Mass Finder Library, and confirmed by a comparison of their retention indices.

#### 4.3.4. Plant Protein Content (%)

Plant samples were taken during harvest. Approximately 10 g of each sample was dried at 65 °C, grounded, and incinerated at 550 °C. The incinerated samples were dissolved in 3.3% HCl and then filtered and diluted to a volume of 100 mL using ultra-pure distilled water. The protein contents (N x 6.25) were based on the Kjeldahl method [69].

### 4.4. Statistical Analysis

The study was arranged as two factors (HA doses and irrigation level) in a randomized complete block design with three replications in both years. The data were subjected to analysis of variance using the MSTAT statistical program. Mean differences were tested with a least significant difference (LSD) test (*p* ≤ 0.05 or *p* ≤ 0.01). 

## 5. Conclusions

Humic acid (HA), irrigation (IR), and soil-mulching (SM) have shown the broad development prospects of basil plants in semi-arid ecological conditions. In this study, the differences between varying HADs and drip IRLs with and without the use of SM were analyzed, and the conclusions were as follows:

(1) The first trial was conducted under various drip IRLs and HADs without the use of SM. The highest mean values of morpho-physiological traits were obtained at IRL 100 and IRL 75 in both years. The mean EOR of basil was highest at IRL 25. FHY and DLY were affected by HAD, and the highest mean values were obtained at HAD 20 and HAD 40.
(2) For the biochemical traits without the use of SM, the mean content of linalool at IRL 100 and IRL 75 and 1,8-cineole at IRL 100 were highest in both years. The mean contents of oxygenated monoterpene hydrocarbons at IRL 100 and IRL 75 and sesquiterpene hydrocarbons at IRL 75 in the first year were higher than the previous IRL.
(3) The second trial was conducted under different drip IRLs and HADs with the use of SM. The mean values of plant height (PH), fresh herb yield (FHY), dry herb yield (DHY), dry leaf yield (DLY), and essential oil yield (EOY) belonging to SM application were higher compared to the plants cultivated without SM. The highest mean values for FHY were obtained at HAD 40 and HAD 20, whereas, without SM, the HA applications were insignificant. SM increased the PH up to +8.8–13.5%, FHY to +11.7–16.7%, and the DLY to +22.5–29.2% compared to the plots without SM. Under SM conditions, higher FHY and DLY were obtained at IRL 50 and higher EOY at IRL 75 and IRL 50. Irrigation at 50% field capacity may decrease water consumption up to 50% compared to the plants cultivated without SM.
(4) Under SM conditions, the ratio of linalool, 1,8-cineole, and (E)-β-bergamotene increased at HAD 40, IRL 25, and IRL 50 which not only preserved the irrigation water up to 50–75% but also produced higher ratios of the main essential oil compounds. With the use of SM, it was also observed that HA worked more efficiently. The increase in the essential oil compounds at IRL 50 and IRL 25 was more pronounced at the dose of HA 40.
(5) The HA used in this study improved FHY and DLY at HAD 20 and HAD 40, the yield values were significantly affected by HAD. Similarly, it increased the main essential oil compounds, especially at HAD 40 and IRL 50 under SM conditions. Because of these results, HA can be considered a plant biostimulant, which was defined by the 2018 Farm Bill (https://bpia.org/solutions-provided-by-biological-products-biostimulants/) and the regulation of (EU) 2019/1009 which defined it as the improvement of yield and quality traits (http://data.europa.eu/eli/reg/2019/1009/oj).

Based on the results above, it seems more reasonable to cultivate basil plants using HAD 40 and IRL 50 to obtain a higher potential of fresh and dry yield and higher amounts of primary essential oil compounds with a soil-mulched drip irrigation system which not only preserves the irrigation water up to 50% but also avoids mechanical and chemical weed control, which is an environmental and economical benefit. The soil-mulched drip irrigation system is easily applicable by local producers, particularly for crops that are harvested several times during a single vegetation.

Eventually, it will be necessary to conduct research with particularly low irrigation levels of a soil-mulched drip irrigation system with basil in different locations and years because the yield and quality under mulching conditions need to be explained for the irrigation level under 25% field capacity. In addition, the methods, frequency, and doses of humic acid applications may be incorporated with various soil mulching materials. 

## Figures and Tables

**Table 1:** Analysis of variance and mean values of the examined morpho-physiological characteristics of *Ocimum basilicum* L. cultivated under different IRL and HAD conditions (2016) without SM.

	PH	NB	FHY	DHY	DLY	CV	EOR	EOY	PR
IRL ^1^									
IRL 100	34.79 a ^#^	11.41	7625.1 a	1124.2 a	652.2 a	46.83	0.48 b	3.1 c	22.64 ab
IRL 75	35.46 a	10.60	7605.9 a	1111.5 a	657.8 a	46.72	0.54 b	3.5 ab	23.20 a
IRL 50	32.85 ab	10.01	6944.4 b	927.8 b	586.9 b	44.45	0.56 b	3.2 bc	21.52 c
IRL 25	31.78 b	9.22	6363.5 b	847.6 b	567.5 b	45.65	0.66 a	3.7 a	21.86 bc
HAD ^2^									
HA 0	34.29	9.77	6852.6 c	966.2	587.0 b	46.13	0.56	3.3	21.91
HA 10	33.82	10.11	6945.3 bc	985.6	604.5 ab	45.13	0.55	3.3	22.21
HA 20	33.43	10.35	7268.3 ab	1015.8	635.3 a	46.46	0.58	3.6	22.40
HA 40	33.35	11.00	7472.7 a	1043.4	637.5 a	45.93	0.54	3.4	22.69
Means	33.72	10.31	7134.72	1002.7	616.1	45.9	0.56	3.4	22.30
IRL	**	ns	**	**	*	ns	**	*	**
HAD	ns ^3^	ns	*	ns	*	ns	ns	ns	ns
HAD × IRL	ns	ns	ns	ns	ns	ns	ns	ns	ns

PH: plant height (cm); NB: number of branches plant^−1^; FHY: fresh herb yield (kg ha^−1^); DHY: dry herb yield (kg ha^−1^); DLY: dry leaf yield (kg ha^−1^); CV: chlorophyll value (SPAD); EOR: essential oil ratio (%); EOY: essential oil yield (L ha^−1^); PR: protein ratio (%) ^1^: irrigation levels, ^2^: humic acid doses, ^3^: non-significant, IRL 100 = 100% FC; IRL 75 = 75% FC; IRL 50 = 50% FC; IRL 25 = 25% FC; HA 0 = 0.0 Lha^−1^; HA 10 = 10.0 Lha^−1^; HA 20 = 20.0 Lha^−1^; HA 40 = 40.0 Lha^−1^; ^#^: means followed by the same letter(s) in each column are not significantly different at * *p* ≤ 0.05; ** *p* ≤ 0.01; SM: soil mulching.

**Table 2:** Analysis of variance and mean values of the examined morpho-physiological characteristics of *Ocimum basilicum* L. cultivated under different IRL and HAD conditions (2017) without SM.

Traits	PH	NB	FHY	DHY	DLY	CV	EOR	EOY	PR
IRL ^1^									
IRL 100	34.07 a ^#^	9.39 a	7139.3 a	979.1 a	566.2 a	46.17 a	0.60 c	3.2	21.81 a
IRL 75	34.94 a	8.94 ab	7064.8 ab	967.0 a	565.0 a	46.07 a	0.61 bc	3.5	21.04 ab
IRL 50	33.48 ab	8.34 b	6274.0 bc	816.2 b	447.5 b	45.19 ab	0.70 ab	3.2	20.21 bc
IRL 25	30.62 b	8.22 b	5999.7 c	763.3 b	420.4 b	44.43 b	0.75 a	3.2	19.24 c
HAD ^2^									
HA 0	33.18	8.69	6511.6	868.8	501.2	44.26	0.69	3.3 ab	20.58
HA 10	32.24	8.73	6505.6	869.4	506.0	45.92	0.70	3.5 a	20.71
HA 20	34.16	8.93	6943.4	928.7	528.6	45.50	0.63	3.3 ab	20.49
HA 40	33.53	8.55	6517.1	858.9	463.3	46.18	0.64	2.9 b	20.52
Means	33.28	8.73	6619.43	881.45	499.78	45.47	0.67	3.25	20.58
IRL	**	*	**	**	**	*	*	ns	**
HAD	ns ^3^	ns	ns	ns	ns	ns	ns	*	ns
HAD × IRL	ns	ns	ns	ns	ns	ns	ns	ns	ns

PH: plant height (cm); NB: number of branches plant^−1^; FHY: fresh herb yield (kg ha^−1^); DHY: dry herb yield (kg ha^−1^); DLY: dry leaf yield (kg ha^−1^); CV: chlorophyll value (SPAD); EOR: essential oil ratio (%); EOY: essential oil yield (L ha^−1^); PR: protein ratio (%) ^1^: irrigation levels, ^2^: humic acid doses, ^3^: non-significant, IRL 100 = 100% FC; IRL 75 = 75% FC; IRL 50 = 50% FC; IRL 25 = 25% FC; HA 0 = 0.0 Lha^−1^; HA 10 = 10.0 Lha^−1^; HA 20 = 20.0 Lha^−1^; HA 40 = 40.0 Lha^−1^; ^#^: means followed by the same letter(s) in each column are not significantly different at * *p* ≤ 0.05; ** *p* ≤ 0.01; SM: soil mulching.

**Table 3:** Essential oil components of *O. basilicum* L. according to different IRLs in 2016 and 2017 without SM.

IRL		IRL 100				IRL 75				
Compounds (%)	RRI	2016	SE (±)	2017	SE (±)	2016	SE (±)	2017	SE (±)	IM
1,8-cineole	1213	3.83	0.94	3.98	0.36	3.15	0.64	2.75	1.24	t_R_,
(E)-β-ocimene	1246	0.80	0.55	0.95	0.26	0.55	0.26	0.80	0.53	t_R_, MS
camphor	1532	0.21	0.02	0.65	0.41	0.30	0.12	0.33	0.26	t_R_, MS
linalool	1553	57.28	4.73	57.53	5.72	56.88	9.01	59.50	5.57	t_R,_ MS
(E)-β-bergamotene	1594	4.08	0.78	5.48	1.58	3.25	1.19	4.50	1.18	MS
*α-guaiene elemene	1597–1607	1.73	0.35	1.45	0.34	2.18	1.09	1.83	0.80	MS
β -caryophyllene	1612	1.73	0.62	1.10	0.43	1.98	1.12	1.73	0.95	t_R_, MS
germacrene D	1726	2.88	0.34	2.20	0.36	3.93	1.87	2.73	1.01	MS
γ-guaiene	1718	1.20	0.14	1.20	0.26	1.63	0.92	1.50	0.62	MS
bicyclogermacrene	1755	1.18	0.10	0.88	0.17	1.10	0.27	1.08	0.45	t_R_, MS
γ-cadinene	1776	1.70	0.22	1.88	0.55	1.68	0.36	1.83	0.72	MS
(Z)-methyl. cinnamate	1980	2.03	0.10	-	-	2.05	0.39	-	-	MS
(E)-methyl cinnamate	2100	9.53	2.18	-	-	9.13	1.52	-	-	MS
cubenole	2080	0.60	0.08	0.80	0.08	0.58	0.10	0.75	0.12	MS
eugenol	2186	3.83	0.68	9.90	1.36	2.53	0.42	9.48	1.52	t_R_, MS
T-cadinol	2187	4.83	0.28	5.00	1.61	4.70	0.74	4.88	1.61	MS
OMH		61.31		62.16		60.33		62.58		
MH		0.80		0,95		0.55		0,80		
SH		14.48		14.19		15.75		15.20		
OSH		5.43		5.80		5.28		5.63		
O		15.39		9.90		13.71		9.48		
Total		97.40		93.00		95.62		93.69		

IRL 100 = 100% FC; IRL 75 = 75% FC; M. cinnamate: methyl cinnamate; RRI: relative retention indices experimentally calculated against *n*-alkanes; %: calculated from FID data; tr: trace (<0.1%); IM: identification method; t_R_: identification based on comparison with co-injected with standards on an HP Innowax column; MS: identified on the basis of computer matching of the mass spectra with those of the in-house Baser Library of Essential Oil Constituents, Adams and Wiley libraries. OMH: oxygenated monoterpene hydrocarbons; MH: monoterpene hydrocarbons; SH: sesquiterpene hydrocarbons; OSH: oxygenated sesquiterpene hydrocarbons; O: others; SM: soil mulching.

**Table 4:** Essential oil components of *O. basilicum* L. according to different IRLs in 2016 and 2017 without SM. (continued).

IRL		IRL 50				IRL 25				
Compounds (%)	RRI	2016	SE (±)	2017	SE (±)	2016	SE (±)	2017	SE (±)	IM
1,8-cineole	1213	3.40	0.86	3.58	0.34	2.58	0.80	2.68	0.6	t_R_, MS
(E)-β-ocimene	1246	0.63	0.21	1.00	0.42	0.55	0.13	0.90	0.41	t_R_, MS
camphor	1532	0.30	0.14	0.68	0.41	0.23	0.10	0.55	0.13	t_R_, MS
linalool	1553	49.55	6.41	54.95	4.02	53.05	4.88	56.70	4.19	t_R_, MS
(E)-β-bergamotene	1594	2.88	0.96	4.48	0.55	2.55	0.44	5.85	1.36	MS
*α-guaiene/elemene	1597–1607	1.25	0.24	1.35	0.37	1.45	0.58	1.83	0.43	MS
β -caryophyllene	1612	1.40	0.72	1.78	1.65	1.20	0.78	1.28	0.38	t_R_, MS
germacrene D	1726	2.35	0.50	2.28	0.62	2.40	0.94	3.13	0.90	MS
γ-guaiene	1718	0.88	0.15	1.18	0.34	1.15	0.48	1.60	0.44	MS
bicyclogermacrene	1755	0.78	0.15	1.08	0.48	0.88	0.10	1.13	0.22	t_R_, MS
γ-cadinene	1776	1.30	0.08	1.65	0.19	1.25	0.24	2.28	0.42	MS
(Z)-methyl cinnamate	1980	1.70	0.18	-	-	3.03	0.41	-	-	MS
(E)-methyl cinnamate	2100	14.75	2.63	-	-	13.63	3.80	-	-	MS
cubenole	2080	0.53	0.05	1.05	0.52	0.48	0.05	0.83	0.43	MS
eugenol	2186	4.78	0.22	10.80	1.21	2.98	1.72	8.48	3.37	t_R_, MS
T-cadinol	2187	3.98	0.53	4.38	0.29	3.65	0.64	5.60	1.12	MS
OMH		53.25		59.20		55.85		59.93		
MH		0.63		1.00		0.55		0.90		
SH		10.83		13.78		10.88		17.08		
OSH		4.50		5.43		4.13		6.43		
O		21,23		10.80		19,63		8.48		
Total		90.42		90.20		91.03		92.81		

IRL 50 = 50% FC; IRL 25 = 25% FC; M. cinnamate: methyl cinnamate. RRI: relative retention indices experimentally calculated against *n*-alkanes; %: calculated from FID data; tr: trace (<0.1%); IM: identification method; t_R_: identification based on comparison with co-injected with standards on an HP Innowax column; MS: identified on the basis of computer matching of the mass spectra with those of the in-house Baser Library of Essential Oil Constituents, Adams and Wiley libraries. OMH: oxygenated monoterpene hydrocarbons; MH: monoterpene hydrocarbons; SH: sesquiterpene hydrocarbons; OSH: oxygenated sesquiterpene hydrocarbons; O: others; SM: soil mulching.

**Table 5:** Analysis of variance of IRL and HAD on some yield traits and quality characters of *O. basilicum* L. under soil mulching and control conditions.

Soil Mulching Plots									
Traits	PH	NB	FHY	DHY	DLY	CA	EOR	EOY	PR
IRL ^1^ HAD ^2^	** *	* ns ^3^	** *	ns ns	* ns	ns *	ns ns	* ns	ns ns
IRL × HAD	*	ns	*	ns	ns	ns	ns	ns	**
**control plots**									
IRL HAD	** ns	ns ns	** *	** ns	* *	ns ns	** ns	* ns	* ns
IRL × HAD	ns	ns	ns	ns	ns	ns	ns	ns	ns

PH: plant height (cm); NB: number of branches plant^−1^; FHY: fresh herb yield (kg ha^−1^); DHY: dry herb yield (kg ha^−1^); DLY: dry leaf yield (kg ha^−1^); CV: chlorophyll value (SPAD); EOR: essential oil ratio (%); EOY: essential oil yield (L ha^−1^); PR: protein ratio (%); ^1^: irrigation levels; ^2^: humic acid doses; ^3^: non-significant; * *p* ≤ 0.05; ** *p* ≤ 0.01.

**Table 6:** The mean values of the morpho-physiological characteristics of *Ocimum basilicum* L. cultivated under different IRL and HAD conditions (the means of the mean values).

	PH	NB	FHY	DHY	DLY	CV	EOR	EOY	PR
CP 2016	33.72	10.31	7134.7	1002.7	616.1	45.9	0.56	3.4	22.30
SM 2016	37.88	10.65	8249.2	1177.6	831.2	44.4	0.48	3.9	22.56
CP 2017	33.28	8.73	6619.4	881.4	499.8	45.5	0.67	3.25	20.58

PH: plant height (cm); NB: number of branches plant ^−1^; FHY: fresh herb yield (kg ha^−1^); DHY: dry herb yield (kg ha^−1^); DLY: dry leaf yield (kg ha^−1^); CV: chlorophyll value (SPAD); EOR: essential oil ratio (%); EOY: essential oil yield (L ha^−1^); PR: protein ratio (%); soil mulching (SM); control plots (CP).

**Table 7:** Essential oil components of *O. basilicum* L. according to different IRLs and HADs under control conditions.

IRL	IRL 100				IRL 75				IRL 50				IRL 25			
HAD (L HA ha^−1^)	0	10	20	40	0	10	20	40	0	10	20	40	0	10	20	40
Compounds (%)																
1,8-cineole	2.5	4.5	3.8	4.5	2.8	2.9	2.8	4.1	3.4	3.0	4.6	2.6	3.2	3.3	2.1	1.7
(E)-β-ocimene	0.4	0.7	1.6	0.5	0.7	0.2	0.5	0.8	0.4	0.5	0.8	0.8	0.4	0.7	0.6	0.5
camphor	0.2	0.2	0.2	0.25	0.4	0.2	0.4	0.2	0.5	0.2	0.3	0.2	0.2	0.3	0.3	0.1
linalool	50.6	60.2	57.3	61.0	52.8	471	59.6	68.0	58.1	50.8	45.2	44.1	56.7	57.7	49.9	47.9
(E)-β-bergamotene	3.1	4.1	4.1	5.0	3.0	2.4	5.0	2.6	3.6	3.8	2.0	2.1	2.3	3.2	2.3	2.4
*α-guaiene/ β-elemene	1.3	1.9	2.1	1.6	1.6	3.8	1.5	1.8	1.1	1.0	1.5	1.4	1.9	2.0	1.0	0.9
Β-caryophyllene	0.9	1.9	1.7	2.4	1.0	3.3	1.1	2.5	0.9	0.7	2.2	1.8	2.1	1.6	0.6	0.5
germacrene D	2.4	3.0	2.9	3.2	2.7	6.7	2.9	3.4	2.3	1.7	2.9	2.5	3.1	3.3	1.8	1.4
γ-guaiene	1.0	1.3	1.2	1.3	1.2	3.0	1.1	1.2	0.7	0.8	1.0	1.0	1.4	1.7	0.8	0.7
bicyclogermacrene	1.1	13	1.2	1.1	1.0	1.5	0.9	1.0	0.6	0.7	0.9	0.9	1.0	0.9	0.8	0.8
γ-cadinene	1.4	1.8	1.7	1.9	1.6	2.2	1.5	1.4	1.4	1.3	1.3	1.2	1.4	1.5	1.1	1.0
(Z)-methyl. cinnamate	2.1	2.0	1.9	2.1	1.7	2	2.6	1.9	1.8	1.5	1.6	1.9	2.9	2.5	3.3	3.4
cubenole	0.5	0.6	0.6	0.7	0.5	0.7	0.6	0.5	0.6	0.5	0.5	0.5	0.5	0.5	0.4	0.5
(E)-methyl. cinnamate	10.5	8.6	12.0	7.0	9.1	10	10.4	7	11.0	15.0	12	10	10.3	10.4	16.4	17.4
eugenol	3.4	4.7	4	3.2	2.4	2.5	2.1	3.1	4.7	5.0	4.5	4.9	4.9	3.7	2.4	0.9
T-cadinol	4.5	4.7	5	5.1	4.3	5.8	4.5	4.2	4.7	3.7	3.5	4.0	3.4	4.6	3.2	3.4

IRL 100 = 100% FC; IRL 75 = 75% FC; IRL 50 = 50 % FC; IRL 25 = 25 % FC; HA 0 = 0.0 Lha^−1^; HA 10 = 10.0 Lha^−1^; HA 20 = 20.0 Lha^−1^; HA 40 = 40.0 Lha^−1^; *α-guaiene/β-elemene.

**Table 8:** Essential oil components of *O. basilicum* L. according to different IRLs and HADs under soil-mulching conditions.

IRL		IRL 100				IRL 75			IRL50					IRL25		
HAD (L HA ha^−1^)	0	10	20	40	0	10	20	40	0	10	20	40	0	10	20	40
Compounds (%)																
1.8-cineole	2.8	3.8	4.4	2.9	2.5	3.0	2.7	1.6	3.5	3.6	2.7	3.8	2.3	4.0	2.5	3.9
linalool	47.7	46.8	60.5	49.5	47.5	60.5	54.3	42.3	60.7	44.7	42.5	62.1	59.3	54.5	53.5	61.6
(E)-β-bergamotene	3.1	4.6	4.8	5.1	3.9	4.2	4.0	3.0	5.2	3.1	1.4	5.4	3.6	3.8	4.4	5.1
*α-guaiene/ β-elemene	1.1	1.6	1.3	1.1	0.9	1.3	1.5	0.8	1.7	1.2	1.0	1.8	1.2	0.8	1.6	1.3
Β-caryophyllene	1.0	1.8	0.8	0.8	0.6	1.1	1.5	0.5	1.2	0.8	0.6	1.4	0.8	0.7	0.9	0.9
germacrene D	2.3	2.7	2.6	1.9	1.5	3.1	2.7	1.6	3.4	2.2	1.7	3.3	2.1	1.6	2.3	2.3
γ-guaiene	0.9	1.8	1.1	1.0	0.7	1.1	1.1	0.7	1.5	1.0	1.3	1.7	1.8	0.6	1.1	1.0
bicyclogermacrene	0.6	0.7	0.9	0.8	0.7	1.1	0.9	0.7	1.3	1.0	0.6	1.3	1.2	0.6	1.0	0.9
γ-cadinene	1.2	1.8	1.5	1.6	1.4	1.6	1.7	1.1	1.8	1.5	0.9	2.1	1.4	1.1	1.6	1.6
(Z)-methyl. cinnamate	2.7	1.7	1.0	1.5	3.0	2.1	1.4	3.3	0.2	1.9	4.4	2.7	-	2.1	1.9	1.5
cubenole	0.5	0.9	0.5	0.6	0.6	0.5	0.6	0.6	0.6	0.5	0.6	0.5	0.6	0.7	0.5	0.7
(E)-methyl. cinnamate	27.2	4.0	9.5	14.9	28.2	7.6	12.0	34.4	2.7	15.3	35.4	0.2	-	17.2	17.7	5.6
eugenol	2.4	6.8	3.1	3.0	1.1	3.3	3.8	1.9	3.7	3.8	4.4	5.2	2.1	2.0	3.1	3.9
T-cadinol	3.2	5.6	4.3	3.9	3.4	4.7	4.4	3.0	4.7	4.4	3.9	5.0	3.6	2.2	4.2	4.3

IRL 100 = 100% FC; IRL 75 = 75% FC; IRL 50 = 50% FC; IRL 25 = 25% FC; HA 0 = 0.0 Lha^−1^; HA 10 = 10.0 Lha^−1^; HA 20 = 20.0 Lha^−1^; HA 40 = 40.0 Lha^−1^; *α-guaiene/β-elemene.

**Table 9:** The average rainfall (mm), temperature (°C), and humidity (%) during the experimental period at Eskisehir in 2016, 2017, and the long term.

	Rainfall (mm)			Humidity (%)			Temperature (°C)		
	2016	2017	Long Term	2016	2017	Long Term	2016	2017	Long Term
March	40.6	16.2	30.3	70.3	68.7	65.1	7.5	7.6	5.1
April	30.6	62.0	42.2	64.5	66.9	62.7	12.9	9.6	9.9
May	44.4	50.8	41.6	74.2	73.0	60.8	14.1	14.4	15.0
June	7.0	44.8	31.1	62.1	73.4	57.2	21.0	19.1	19.2
July	12.0	13.4	12.4	58.3	59.5	53.0	22.8	23.1	22.2
August	26.4	31.4	13.0	66.0	67.3	54.6	22.8	22.0	22.0
September	31.1	3.0	18.1	67.1	57.0	58.2	17.8	19.6	17.3
Total/mean	192.1	221.6	188.7	66.0	66.5	58.8	16.9	16.4	15.8

**Table 10:** Soil characteristics of the research area in 2016 and 2017.

	2016	2017
EC(dS/m)	0.060	0.045
pH	7.78	8.14
CaCO_3_ (%)	6.7	5.9
Organic matter (%)	2.80	0.31
Available P_2_O_5_ (kg ha^−1^)	0.736	0.542
Available K_2_O (kg ha^−1^)	24.10	48.11
Fe (mg kg^−1^)	4.87	2.82
Zn (mg kg^−1^)	0.64	0.51
Mn (mg kg^−1^)	28.98	15.30
Cu (mg kg^−1^)	2.45	2.38

**Table 11:** Irrigation water quality in 2016 and 2017.

	2016	2017
Cations	meq/lt	meq/lt
Sodium	1.48	1.27
Potassium	0.13	0.30
Calcium	3.54	3.33
Magnesium	4.46	5.27
Total	9.61	10.16
Anions	meq/lt	meq/lt
Carbonate	0.00	0.00
Bicarbonate	5.40	5.00
Chlorine	0.20	0.20
Sulfate	4.01	4.96
Total	9.61	10.16
pH of water	7.98	7.83
EC (dS/m at 25 °C)	0.803	0.836
Hardness (French hardness)	0.74	0.61

**Table 12:** Some properties of HA used in the study.

Properties	Unit	Values
pH	-	11.8
EC	(dS m^−1^)	5.6
HA + FA	(%)	12.0
Organic Matter	(%)	5.0
K	(%)	1.60
Na	(%)	0.49
N	(%)	0.23
Ca	(mg kg^−1^)	5228
Mg	(mg kg^−1^)	680
Fe	(mg kg^−1^)	550
P	(mg kg^−1^)	72
Cu	(mg kg^−1^)	2.4
Mn	(mg kg^−1^)	6.2
Zn	(mg kg^−1^)	2.4

HA + FA: humic acid + fulvic acid.

## Data Availability

All new research data were presented in this paper.

## Supplementary Materials

The following supporting information can be downloaded at: https://www.mdpi.com/article/10.3390/plants12071522/s1, Table S1. Analysis of variance and mean values of the examined charac-teristics of Ocimum basilicum L. cultivated under different IRL and HAD conditions (2016) with SM.
