# Supplementary material for: The Effect of Irrigation and Humic Acid on the Plant Yield and Quality of Sweet Basil (Ocimum basilicum L.) with Mulching Application under Semi-Arid Ecological Conditions
Source: Plants (Basel). 2023 Mar 31;12(7):1522. doi: 10.3390/plants12071522 (PMC10097155; doi:10.3390/plants12071522)
Supplement: Supplementary file 1 [file plants-12-01522-s001.zip › plants-2169577-supplementary.pdf]

Supplementary Table S1. Analysis of variance and mean values of the examined characteristics of *Ocimum basilicum* L. cultivated under different IRL and HAD conditions (2016) with SM.

	PH	NB	FHY	DHY	DLY	CV	EOR	EOY	PR
IRL									
IR 100	38.16 a	11.31 a	8639.0 a	1264.1	880.8 a	43.73	0.45	3.9 ab	22.15
IR 75	39.22 a	10.93 a	8726.7 a	1236.5	881.7 a	42.89	0.53	4.6 a	22.48
IR 50	38.00 a	10.81 a	8335.2 ab	1178.0	829.4 a	45.75	0.49	4.0 a	23.02
IR 25	36.14 b	9.54 b	7296.3 b	1031.7	732.9 b	45.20	0.45	3.3 b	22.61
HAD									
HA 0	37.55 b	10.58	7931.4 b	1146.3	791.4	45.19 ab	0.47	3.8	22.65
HA 10	36.80 b	10.73	8165.8 b	1164.8	819.2	43.24 c	0.51	4.1	22.69
HA 20	38.10 ab	10.74	8319.5 ab	1168.8	830.5	45.42 a	0.46	3.9	22.43
HA 40	39.07 a	10.54	8580.5 a	1230.5	883.6	43.73 bc	0.47	4.1	22.48
Means	37.88	10.65	8249.2	1177.6	831.2	44.39	0.48	3.9	22.56
			**ANOVA**						
IRL[1]	**	*	**	ns	*	ns	ns	*	ns
HAD[2]	*	ns[3]	*	ns	ns	*	ns	ns	ns
HADx IRL	*	ns	*	ns	ns	ns	ns	ns	**

PH: Plant Height (cm); NB: Number of Branches plant $^{-1}$; FHY: Fresh Herb Yield (kg ha^{-1}); DHY: Dry Herb Yield (kg ha^{-1}); DLY: Dry Leaf Yield (kg ha $^{-1}$); CV: Chlorophyll Value (SPAD); EOR: Essential Oil Ratio (%); EOY: Essential Oil Yield (L ha $^{-1}$); PR: Protein Ratio (%) [1]: Irrigation Levels, [2]: Humic Acid Doses, [3]:non-significant, IRL 100=100 % FC; IRL 75=75 % FC; IRL 50=50 % FC; IRL 25=25 % FC; HA 0=0.0 Lha$^{-1;}$; HA 10=10.0 Lha^{-1} ; HA 20=20.0 Lha^{-1} ;HA 40=40.0 Lha^{-1} [3]: non-significant; *$p \leq 0.05$; **$p \leq 0.01$.
